# Effectiveness of enhanced cognitive behavioral therapy (CBT-E) for eating disorders: study protocol for a randomized controlled trial

**DOI:** 10.1186/s13063-016-1716-3

**Published:** 2016-12-03

**Authors:** Martie de Jong, Kees Korrelboom, Iris van der Meer, Mathijs Deen, Hans W. Hoek, Philip Spinhoven

**Affiliations:** 1 Center for Eating Disorders – PsyQ, part of Parnassia Psychiatric Institute, The Hague, The Netherlands; 2 Parnassia Psychiatric Institute, The Hague, The Netherlands; 3 Department of Medical and Clinical Psychology, Tilburg University, Tilburg, The Netherlands; 4 Leiden University, Institute of Psychology, Methodology and Statistics Unit, Leiden, The Netherlands; 5 Department of Psychiatry, University of Groningen, University Medical Center Groningen, Groningen, The Netherlands; 6 Department of Epidemiology, Columbia University, Mailman School of Public Health, New York, NY USA; 7 Leiden University, Institute of Psychology, Leiden, The Netherlands; 8 Department of Psychiatry, Leiden University Medical Center, Leiden, The Netherlands

**Keywords:** Eating disorders, Transdiagnostic treatment, Cognitive-behavioral therapy, CBT-E, Treatment outcome, Cost-effectiveness, RCT

## Abstract

**Background:**

While eating disorder not otherwise specified (EDNOS) is the most common eating disorder (ED) diagnosis in routine clinical practice, no specific treatment methods for this diagnosis have yet been developed and studied. Enhanced cognitive behavioral therapy (CBT-E) has been described and put to the test as a transdiagnostic treatment protocol for all EDs, including EDNOS. Initial research in the UK suggests that CBT-E is more effective for EDs, especially bulimia nervosa (BN) and EDNOS, than the earlier version of CBT. These positive results of CBT-E have to be replicated in more detail, preferably by independent researchers in different countries. Being the first Dutch study into CBT-E, the results from this national multicenter study – on three sites specialized in EDs – will deliver important information about the effectiveness of CBT-E in several domains of ED pathology, while providing input for the upcoming update of the Dutch Multidisciplinary Guideline for the Treatment of Eating Disorders.

**Methods/design:**

A multicenter randomized controlled trial will be conducted. One hundred and thirty-two adult outpatients (aged 18 years and older) with an ED diagnosis and a Body Mass index (BMI) of between 17.5 and 40 will be randomly allocated to the control or the intervention group. Subjects in the control group will receive Treatment as Usual (standard outpatient treatment provided at the participating sites). Subjects in the intervention group will receive 20 sessions of CBT-E in 20 weeks. The design is a 2 (group) × 5 (time) repeated measures factorial design in which neither therapists nor patients will be blinded for treatment allocation. The primary outcome measure is recovery from the ED. Secondary outcome measures include ED psychopathology, common mental disorders, anxiety and depressive symptoms, health-related quality of life, health care use and productivity loss. Self-esteem, perfectionism and interpersonal problems will be examined as putative predictors and mediators of the effect of treatment. Also, an economic evaluation from a societal perspective will be undertaken. All relevant effects, direct and indirect costs will be included. Utility scores will measure the effects. Measurements will take place at pretreatment, 6 weeks, 20 weeks, 40 weeks and 80 weeks.

**Discussion:**

This effectiveness study into CBT-E has the aim of broadening the scope and generalizability of former studies. If CBT-E appears to be at least as effective as traditional diagnosis-specific treatments for a broad range of ED patients, training in one protocol would be sufficient for clinicians to treat patients with different kinds of EDs. It gives the opportunity to offer treatment for a severe mental disorder with fewer resources, thereby increasing the accessibility of specialized care for patients with an ED.

**Trial registration:**

Netherlands Trial Register, NTR4485. Registered on 2 April 2014.

**Electronic supplementary material:**

The online version of this article (doi:10.1186/s13063-016-1716-3) contains supplementary material, which is available to authorized users.

## Background

Eating disorders (EDs) are severe mental disorders, which typically begin in adolescence [1–4]. In the fourth edition of the *Diagnostic and Statistical Manual of Mental Disorders* (DSM-IV) [5] three EDs are recognized: anorexia nervosa (AN), bulimia nervosa (BN) and a residual diagnostic category called eating disorder not otherwise specified (EDNOS) including binge eating disorder (BED). In the new edition, DSM-5 [6], BED has been added as a new official diagnosis [7]. Prior to the official recognition of BED as a specific DSM-5 ED, several studies into the efficacy of specific BED interventions have been performed [8], utilizing DSM-IV research criteria. In DSM-5, the remaining EDs from the DSM-IV EDNOS category have been redefined into two categories: other specified feeding or eating disorder (OSFED) and unspecified feeding or eating disorder (USFED).

These developments have complicated direct comparisons between research data on the DSM-IV EDNOS and the DSM-5 BED, OSFED and USFED categories.

The effectiveness research on EDs has focused on BN and, more recently, BED. Studies of good quality on DSM-IV EDNOS (with the exception of BED) and AN are limited. This paucity of research on ED symptoms summarized in the DSM-IV EDNOS category is a serious problem, as EDNOS has been the most common ED diagnosis (50–77%) in routine clinical practice [9, 10], and is responsible for severe morbidity, loss of quality of life and even an annual mortality rate of 3.3 per 1000 person years [11]. General health care utilization among this group of patients is high [12], and about 62% are referred to mental health care by their GPs [13].

EDNOS often refers to ED psychopathology that does not meet the full diagnostic criteria of one of the specific EDs (i.e., AN or BN). Examples are: (in women) all symptoms of AN except amenorrhea, and compensatory behavior by individuals of normal weight after eating small amounts of food. Although the use of DSM-5 criteria effectively reduces the frequency of the residual diagnosis EDNOS (for example, by lowering the threshold for AN and BN and adding BED as a specified ED), the magnitude of this reduction varies across studies [14].

The three most prominent guidelines – from the UK National Institute for Health and Care Excellence in 2004 [15], from the American Psychiatric Association in 2006 [16] and the most recent from the Royal Australian and New Zealand College of Psychiatrists in 2014 [17] – recommend cognitive behavioral therapy (CBT) as the psychological treatment of first choice, specifically for BN and BED. Specific treatment recommendations for AN are less forthcoming due to the paucity of positive outcome data in this area. These recommendations concur with those from the Dutch Multidisciplinary Guideline for the Treatment of Eating Disorders in 2006 [18].

Fairburn [19] developed a relatively short transdiagnostic CBT, CBT-E(nhanced), designed to be suitable for the full range of ED diagnoses. CBT-E is based upon the transdiagnostic theory of the maintenance of EDs, in which it is assumed that most of the mechanisms involved in the persistence of EDs are common to all three EDs, rather than being specific to each diagnostic group separately [20]. According to Fairburn and colleagues, EDs have more similarities than differences, especially the core psychopathology (over-evaluation of shape and weight) and expression in attitudes and behavior (dietary restriction, dietary rules, binges, self-induced vomiting, etc.). CBT-E, the enhanced version of CBT, uses new strategies and procedures to address mechanisms that are central to the maintenance of all EDs, including the diversity of ED psychopathology that until recently comprised the DSM-IV EDNOS category. CBT-E is characterized by increased focus on engagement, greater emphasis on the modification of concerns about shape and weight, and the development of skills to deal with setbacks. Regardless of ED diagnosis, CBT-E is designed as an individualized and “modular” form of treatment, in which specific modules may be directed at the particular maintaining mechanisms operating in the individual patient’s case.

There are two forms of CBT-E: a focused form (CBT-Ef) that targets ED psychopathology exclusively (e.g., procedures directed at over-evaluation of shape and weight), and a more complex broad form (CBT-Eb) that also addresses additional problems that appear to maintain EDs or complicate their treatment. Fairburn et al. [21] state that additional mechanisms such as clinical perfectionism, low self-esteem and interpersonal problems maintain the ED psychopathology and thereby obstruct change during the treatment with CBT-E. The broad version of CBT-E was designed to focus on these mechanisms. For both versions of CBT-E two variants of intensity have been developed: 20 sessions in 20 weeks for the patients who are not significantly underweight (Body Mass Index (BMI) above 17.5), and 40 sessions in 40 weeks for the patients who are significantly underweight (BMI below 17.5).

First studies have found CBT-E to be more efficacious than other psychological approaches [22–24]. It seems feasible to treat a broad range of ED patients with CBT-E, but more evidence is required according to a recent meta-analysis [25] and the most recent guidelines [17]. The current study will not only evaluate the effectiveness of CBT-E in terms of the reduction of ED psychopathology and additional comorbid psychopathology and enhancement of quality of life and health status, but also the cost-effectiveness of CBT-E relative to regular ED therapy. Treatment as Usual (TAU) for patients with an ED varies per ED category. For BN and BED there are well-described and evaluated CBT protocols [26, 27]. For AN and EDNOS (except BED) evidence-based treatment protocols are lacking and treatments vary greatly. There are no empirical data about duration, intensity and costs of regular therapy for EDs in The Netherlands. However, consulted independent ED experts in the Netherlands and Belgium have estimated that TAU for EDs is probably more intensive, long-term and less effective than CBT-E. Therefore, we expect CBT-E to be more cost-effective compared to regular treatment.

If CBT-E indeed appears to be at least as effective as traditional diagnosis-specific treatments for a broad range of ED patients, this unified transdiagnostic approach for all EDs would give the opportunity to offer treatment for a severe mental disorder with fewer resources and, therefore, increase the accessibility of an evidence-based treatment for patients with an ED.

In this study we only use the focused version (CBT-Ef) for patients with a BMI above 17.5. BMI above 17.5 is considered by Fairburn as the critical limit for the 20-session CBT-E variant. Additional measurements on top of the outcome measures and those for quality of life and health status involve perfectionism, self-esteem and interpersonal problems. These are believed to be possible clinical and research indications for obstruction in change and progress. Measurements will be taken before, during and after treatment to explore the predictive and mediating effects on treatment outcome.

## Methods/design

### Design

**Fig. 1:**
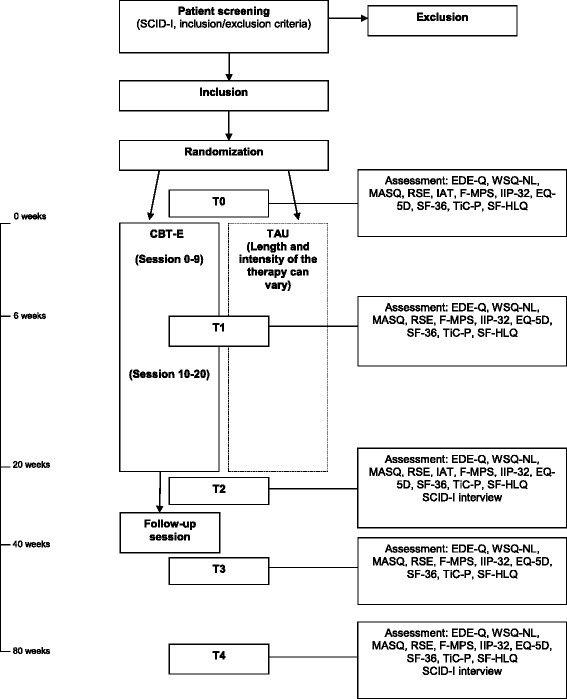
Proposed flow of participants. *T0* baseline, *T1* week 6, *T2* end of treatment week 20, *T3* follow-up week 40, *T4* follow-up week 80. *SCID-I* Structured Clinical Interview for DSM Axis-I disorders, *EDE-Q* The Eating Disorder Examination-Questionnaire, *WSQ* Web Screening Questionnaire for common mental disorders, *MASQ* Mood and Anxiety Questionnaire, *EQ-5D* EuroQoL five dimensions questionnaire, *SF-36* Short Form Health Survey, *TiC-P* Trimbos/iMTA Questionnaire for Costs associated with Psychiatric Illness, *RSE* Rosenberg Self-Esteem Scale, *IAT* Implicit Association Test Self-Esteem. *F-MPS* Frost Multidimensional Perfectionism Scale, *IIP-32* Inventory of Interpersonal Problems

We will execute a multicenter randomized controlled trial (RCT) with two equal-sized parallel groups at three specialized ED treatment centers from three regions in The Netherlands. Participants will be randomized into two groups (CBT-E versus TAU) stratified by ED center and type of ED. Measurements will take place at pretreatment, 6 weeks, 20 weeks, 40 weeks and 80 weeks, resulting in a 2 (group) × 5 (time) repeated measures factorial design. For an overview of the proposed flow of participants, see Fig. 1. The present study protocol was written in accordance with the Standard Protocol Items: Recommendations for Interventional Trials (SPIRIT) [28]; copies of the SPIRIT Checklist and figure have been included in Additional files 1 and 2.

### Participants

Participants will consist of 132 adult outpatients aged from 18 years with an ED diagnosis according to DSM-5 and a BMI of between 17.5 and 40. They are recruited at the participating mental health centers: PsyQ/Parnassia Psychiatric Institute in The Hague will include 60 patients and PsyQ/Lentis Psychiatric Institute in Groningen and Rintveld/Altrecht Mental Health Institute in Zeist both 36 patients.

### Inclusion criteria

In order to be eligible to participate in this study, a participant must meet all of the following criteria:

1. Adult outpatients (from age 18 years) with an ED diagnosis: AN, BN, BED, OSFED (EDNOS), according to an adapted version of the SCID-I (see “Measurement” section) *and* a BMI of between17.5 and 40
2. Provision of informed consent
3. Ability to understand Dutch (speaking, listening, reading)

### Exclusion criteria

A potential participant who meets any of the following criteria will be excluded from participation in this study:

1. Prior treatment that closely resembles CBT-E or another evidence-based intervention for eating pathology in the past 2 years
2. A severe Axis-I or -II psychiatric disorder or other psychosocial circumstances that require priority of clinical attention and other support and, therefore, impedes immediate treatment of the ED (e.g., psychoses, addiction, suicidality, homelessness)
3. Receiving ongoing psychiatric treatment (except for antidepressant medication)
4. Intellectual disability
5. Medical instability or pregnancy
6. Not available over the coming 20 weeks

### Procedure

Each patient will be recruited at the site at which they were referred for treatment. All assessors have been trained in the adjusted SCID-I ED section which will be used to diagnose Axis-I ED. During intake potential participants receive information about the research (treatment conditions, procedure, randomization process, confidentiality) from the local assessors. The assessment staff of each site will decide whether a patient meets the inclusion or exclusion criteria and whether they are definitely eligible for the study. If this is considered to be the case, the research assistant (who is located at the logistic center of the study, PsyQ The Hague) will, for each site, randomly assign the patient to TAU or CBT-E and send an email (in attachment) to the assessor. Randomization is done by making use of a random allocation program, stratified by center and type of ED. Participation will be discussed with the patient during a second appointment with the assessor. If the patient is willing to participate and has signed the Informed Consent Form, the assessor will open the email to inform the patient of the condition they are assigned to. Data will be obtained mainly by online questionnaires, with exception of the SCID-I ED section, which will be conducted by telephone, and the IAT computer task which will be conducted on a stand-alone computer. Prior to the first treatment session, patients will be asked to fill out the online questionnaires and complete the IAT computer task. After 6, 20, 40 and 80 weeks the online questionnaires are obtained. After 20 and 80 weeks the SCID-I will be repeated. After 20 weeks the IAT task will be repeated (for an overview of the assessments see Fig. 1). Participants who do not complete the online questionnaires within 1 week will be contacted by means of personalized emails and/or telephone calls. If they decide to discontinue study participation, efforts will be made to retain them in the trial, while respecting their right to withdraw from participation at any time without further consequences. Patients will not receive any monetary compensation for their involvement, but treatment will be delivered free of charge.

### Study conditions

*CBT-E*: a transdiagnostic 20-session version of CBT, CBT-E(nhanced). In this study we use the focused version for patients with a BMI above 17.5, designed to be suitable for the full range of ED diagnoses. CBT-E is a treatment for ED psychopathology, rather than for a specific ED diagnosis. The strategy underpinning CBT-E is to construct a transdiagnostic formulation (or set of hypotheses) of the processes that are maintaining the patient’s psychopathology and to use this formulation to identify the features that need to be targeted in treatment. This formulation is constructed at the beginning of treatment, but will be revised ,if needed, during therapy. In this way a tailor-made treatment is created.

Stage 1 (sessions 1–7) is an intensive initial stage, with appointments twice a week. The therapist and the patient together set up the formulation of the underlying maintaining factors, which will be used as a base for the remainder of the treatment. The aims of this stage are to engage the patient in treatment.

Stage 2 (sessions 8–9) are weekly appointments. This stage is a brief stage in which the therapist and patient take stock, review progress, identify any emerging barriers to change, modify the formulation and plan stage 3. This stage is important to identify problems with the therapy, to remove barriers and adjust treatment if needed. After stage 2 the treatment will become more personalized.

Stage 3, (sessions 10–17) is the main body of treatment. There are eight weekly appointments. The aim is to address the main mechanisms that are supposed to maintain the patient’s ED. How this is done precisely varies from patient to patient. The therapist can choose to pay attention to one or more defined maintaining factors.

Stage 4 (sessions 18–20) is the final stage of treatment and the focus shifts to the future. The appointments are scheduled at 2-week intervals. There are two aims: the first one is to ensure that the changes are maintained (over the subsequent 20 weeks until a review appointment is held), and the second one is to minimize the risk of relapse in the long-term.

After 20 weeks there is a review session. The most important aim in this session is to review what has been learned and achieved during treatment and what risk factors are to be taken into account when therapy has ended.

*TAU*: the usual treatment given at the participating treatment sites is in general based on CBT, individually or in a group with elements of existing CBT treatment protocols [26, 27]. Depending on the site’s treatment policy, this may vary from low-intensity care (weekly sessions) to high-intensity care. This high-intensity care consists of two group sessions a day for 2 days of the week, sometimes supplemented with individual sessions due to coexisting psychopathology. Most of the times more than one discipline (psychologist, dietitian, psychiatrist) is involved in applying the treatment. The type of treatment provided is registered.

### Selection and training of therapists

All CBT-E therapists are psychologists/psychiatrists or registered nurses/social workers (*n* = 10). All have at least 2 years of experience as a therapist in the field of EDs and have been working for at least 2 years according to CBT principles. All CBT-E therapists in the participating centers were trained as a group by Christopher G. Fairburn and had 20 supervision sessions through videoconferencing from Zafra Cooper. A Dutch treatment manual was developed and will be used by all CBT-E participating therapists. All participating CBT-E therapists have treated at least three ED patients with CBT-E under supervision before entering the trial.

All TAU therapists are psychologists/psychiatrists or registered nurses/social workers who have at least 2 years of experience as therapists in the field of EDs. TAU did not include training or supervision of the therapists. TAU therapists have regularly standard, local collegial consultation.

The treatment integrity in CBT-E will be evaluated by recording all CBT-E sessions. Two audiotaped sessions of every CBT-E patient will be randomly selected (from, respectively, stages 1/2 and stages 3/4) and the use of specific therapeutic interventions according to the treatment manual will be scored on several 7-point Likert scales. The first 20 audiotapes will be double rated to assess interrater reliability. Evaluation of the CBT-E sessions will be executed by psychologists who are familiar with the treatment protocol (trained through the online training in CBT-E developed by Fairburn), by ticking on prearranged checklists whether all due aspects of specific therapy stages have been handled adequately by the therapist.

### Objectives

The primary objective of this study is to assess whether CBT-E is more optimal in terms of a higher percentage of recovery from EDs compared to TAU. The secondary objectives are to assess whether CBT-E is more effective in (1) reducing important aspects of ED psychopathology, (2) reducing indications for the presence of comorbid psychopathological conditions and additionally comorbid symptoms of anxiety and depression, (3) improving health-related quality of life and (4) effectuating a better cost-effectiveness, compared to TAU.

Moreover, self-esteem, perfectionism and interpersonal problems are repeatedly measured during this RCT to examine their possible predictive and mediating effects on treatment outcome.

### Measurements

#### Screening and primary treatment outcome

*Structured Clinical Interview for DSM Axis-I Disorders (SCID-I).* The SCID-I [29, 30] will be used to assess the presence of an ED. Only the section about EDs will be administered. Because the SCID-I only covers AN, BN, and EDNOS-BED, skip rules were changed or omitted and parts of the *Eating Disorder Examination (EDE)* [31] were added in order to diagnose DSM-5 AN, BN, BED and OSFED [32]. The interview will be used both to obtain a DSM-5 diagnosis for inclusion and as a treatment outcome measure at T2 (at the end of treatment, 20 weeks after treatment has started) and T4 (80 weeks after the start of treatment). Several studies found moderate to excellent interrater agreement for determining the presence of Axis-I disorders using the original SCID-I [33] and good test-retest reliability [34, 35].

#### Secondary study parameters

*The Eating Disorder Examination-Questionnaire (EDE-Q)* [36, 37]. This questionnaire is a self-report measure that was adapted from the interview-based EDE [38] and measures ED pathology. It consists of 36 items that are scored on a 7-point scale. The total score is used as an indicator for the level of ED pathology, with a higher score denoting more pathology. Good concurrent validity [39–42], discriminant validity [43] and acceptable criterion validity [42] have been demonstrated with adults. Moreover, the EDE-Q has been found to have good internal consistency and test-retest reliability in adults [44]. In studies comparing the EDE interview and EDE-Questionnaire the overall correlation coefficient ranged from .68 to .76. In general, participants obtain higher scores in the questionnaire than in the interview mode of administration [45, 46].

*Web Screening Questionnaire for common mental disorders (WSQ)* [47]. This self-report screening instrument will be used to screen for Axis-I disorders. It is a short screening instrument for depressive disorder, alcohol abuse/dependence, generalized anxiety disorder (GAD), post-traumatic stress disorder (PTSD), social phobia, panic disorder, agoraphobia, specific phobia, and obsessive compulsive disorder (OCD). The questionnaire consists of 15 questions. The sensitivity of the WSQ is 0.72–1.00 and specificity is 0.44–0.77 [47].

*Mood and Anxiety Symptom Questionnaire (MASQ)* [48]. The MASQ is a self-report questionnaire to assess the severity of symptoms of anxiety and depression. It is based on the tripartite model of anxiety and depression symptoms, which can be separated into three groups: global discomfort (anxiety and depression), anhedonia (specific for depression) and physiological hyperarousal (specific for anxiety). The questionnaire consists of 90 items, with an answering scale from 1 to 5 (“not” to “very much”). The scores of the subscales are measured by summating the scores of the items of the subscales. The subscales have sufficient discriminant validity, especially the depression scales [49–51]. The subscales seem to have sufficient internal consistency [48].

*EuroQoL five dimensions questionnaire (EQ-5D*) [52]. The EQ-5D aims to measure health-related quality of life. The EQ-5D is a short questionnaire that consists of five questions with three answer levels, reflecting “no problem”, “some problem” and “extreme problem” in relation to specific dimensions (i.e., mobility, self-care, usual activity, pain and mood). In addition the EQ-5D also includes a Visual Analog Scale (VAS) to value the respondent’s health state, labeled from “best imaginable health” (100) to “worst imaginable health” (0). The EQ-5D can be used to assess sociodemographic differences in health status. Research provides support for the validity of the EQ-5D as a measure for health status [53].

*Short Form Health Survey (SF-36)* [54]*.* We will use the SF-36 to assess health-related quality of life and health status. The SF-36 was developed for a wide range of chronic diseases [54]. It is a multidimensional instrument, with 36 questions to measure eight dimensions: physical functioning, social functioning, role limitations (physical and emotional), mental health, vitality, pain, general health perception and health change. The scores per dimension will be transformed to a scale from 0 to 100 and a higher score denotes a better health status. The Dutch translation has good reliability (Cronbach’s alpha coefficients above .70) and validity [55].

*Trimbos/iMTA Questionnaire for Costs associated with Psychiatric Illness (TiC-P)*, *including the Short Form – Health and Labour Questionnaire (SF-HLQ)* [56]. The Tic-P is a validated tool commonly applied in economic evaluations of treatments in mental health care. The TiC-P is a paper and pencil self-report questionnaire that consists of two parts. The first part obtains information about the volume of health care consumption (direct costs) and the production losses relative to the health problem in question (indirect costs), and some general questions. The second part of the TiC-P, which measures the indirect costs, is the SF-HLQ. The SF-HLQ, an abbreviated version of the HLQ, is a generic and validated measurement instrument to collect data on productivity losses related to health problems in individuals with paid or unpaid work [56]. By multiplying the volumes by the cost prices, it is possible to calculate the costs [57].

#### Putative predictors/mediators

*The Rosenberg Self-Esteem Scale (RSE).* The RSE is a widely-used 10-item Likert scale to measure self-esteem. Items are answered on a 4-point scale – from “strongly agree” to “strongly disagree” – measuring positive and negative feelings towards the self [58]. The Dutch version of the RSE is found to be a one-dimensional scale with high internal consistency (Cronbach’s alpha of 0.89) and congruent validity [59].

*Implicit Association Test Self-Esteem (IAT)* [60]. The IAT will be used to assess implicit self-esteem. The IAT is a computer-administered task, which measures the automatic associations between concepts. The IAT is based on a double discrimination task in which participants are asked to assign single stimuli as fast as possible to a given pair of target categories. The internal consistency has an average score of 0.70 [60].

*Frost Multidimensional Perfectionism Scale (F-MPS)* [61]. We will use the F-MPS to assess perfectionism. The scale contains 35 questions with a 5-point Likert scale from “strongly disagree” to “strongly agree.” When the scale was developed it measured six subscales of perfectionism. It is regarded as internally consistent, reliable over time and displays sound concurrent validity [61, 62]. However, in practical applications, the six-factor structure appeared to be unstable and an alternate four-factor structure was proposed by several others [63, 64].

*Inventory of Interpersonal Problems (IIP-32)* [65]. The IIP is a self-report questionnaire that measures the interpersonal problems that people experience. The instrument was first developed as a 127-item questionnaire on the basis of a list of common interpersonal difficulties raised by persons seeking psychotherapy. The 64-item version was created by Alden et al. [66] specifically to provide a circumplex measure (originally called the IIP-C). For this research we will use the shorter 32-item version (IIP-32), which was developed with the aim of providing a more rapid assessment with a good reliability and validity [67]. All items are rated on a 5-point Likert scale ranging from 0 (“not at all”) to 4 (“extremely”). The questionnaire has good internal consistency, the coefficient alpha for the scales of the IIP-32 are above 0.70 [65].

### Sample size

In order to detect an absolute difference in recovery rate from ED of 25% (CBT-E: 50% versus TAU: 25%), a sample size of 66 patients per treatment condition is required to provide 80% power at two-sided *p* < 0.05 (intention-to-treat analysis). This means that at least 132 patients are needed for this study.

### Randomization, treatment allocation and blinding

Randomization takes place after screening of the inclusion/exclusion criteria and signing of informed consent. The research assistant will randomly assign the participating patients to CBT-E or TAU, stratified by center and type of ED (AN, BN, BED, OSFED). Within each of the twelve strata, a research assistant will randomize participants using a permuted block design. Given the nature of the psychological treatment neither the therapists nor participants can be blinded for the delivered treatment.

### Data management and storage

All study-related data and other study material will be stored securely at the study site (PsyQ The Hague). Participant information and study data will be kept in locked cabinets in areas with limited public access. After obtaining informed consent, participants will be allocated a unique code. The file that links participants to their codes is stored on a secure server hosted by PsyQ and is only accessible by the researcher and research assistant. Any study material concerning participant information will not be released outside the study without written permission from the participant. Online questionnaires will be collected using an authorized SurveyMonkey account and downloaded and added to the database. The SurveyMonkey security and privacy statements for Internet security and handling personal information and data can be found at, respectively, https://www.surveymonkey.com/mp/policy/security/ and https://www.surveymonkey.com/mp/policy/privacy-policy/.

Data collected on paper (SCID-I), will be manually entered into a database. Data collected by the IAT computer task will be transcripted and added to the database. Data integrity will be enforced through several ways, including valid values, range checks and consistency checks. The master database will be held on a secure server hosted by PsyQ, only accessible for authorized personnel involved in the trial. All obtained data and administrative forms (e.g., informed consent) will be stored in accordance with the data storage protocol for 15 years.

### Statistical analysis

All participants who are randomized will be included in the comparison and analyzed according to their randomized allocation (intent-to-treat analysis). Wherever possible, we will continue to collect follow-up data from participants after any dropout from treatment or from the study in order to keep the dataset as complete as possible. In addition, baseline differences in study completers and dropouts will be analyzed with *t* tests for independent samples or chi-square analyses if appropriate. Moreover, we will perform a per-protocol analysis by including only those participants who completed at least 70% of the scheduled therapy sessions. All analyses will be carried out using SPSS 23 [68].

#### Primary study parameter(s)

To test the hypothesis that CBT-E is more effective than TAU, post-treatment differences in recovery rate (based on SCID-I diagnosis) between conditions will be analyzed with chi-square analysis. Using logistic regression analysis with recovery at post treatment as outcome and treatment condition and baseline EDE-Q scores as predictors, whether differences in recovery rate between conditions are independent of severity of ED pathology at pre treatment will also be investigated. Moreover, the course of scores from pre, mid and post treatment to follow-ups I and II on the EDE-Q will be analyzed with multilevel analysis (MLA). MLA is especially suitable to analyze repeated measures data because it takes into account the dependencies among observations nested within individuals. Another advantage of this methodology is its ability to handle missing data, a problem often occurring in longitudinal research [69]. The data have a three-level hierarchical (multilevel) structure: repeated measures at the first level, individuals at the second level and treatment at the third level. Besides main effects for treatment and time, whether groups differ in their course of EDE-Q scores will be investigated by including a treatment × time interaction term. Differences between treatment centers will be investigated likewise.

#### Secondary study parameter(s); indirect clinical effectiveness

Three-level MLA will also be used to study the relative efficacy of CBT-E versus TAU in reducing scores on the secondary outcome measures.

#### Putative mediators

To test the hypothesis that the effects of the CBT-E/TAU on the EDE-Q scores are mediated by the putative mediators investigated (i.e., self-esteem, perfectionism and interpersonal problems), first, standardized residualized gain scores are calculated by removing the portion of mid-treatment scores on the mediators that can be predicted linearly by corresponding pre-treatment scores and the portion of post-treatment EDE-Q scores that can be predicted linearly by mid-treatment EDE-Q scores. Next, following the analytic steps outlined by Baron and Kenny [70] and Kraemer et al. [71] we will test the significance of the following paths using linear regression analyses: path a: the independent variable (i.e., CBT-E/TAU) must affect the mediator (i.e., pre- to mid-residualized change scores for self-esteem, perfectionism or interpersonal problems); path b: the mediator must affect the dependent variable (i.e., mid- to post-residualized EDE-Q change scores); path c: the independent variable must affect the dependent variable; and path c’: the *direct* effects of treatment on the dependent variable must be meaningfully reduced when including a hypothesized mediator in the model. When early process changes predict later outcome changes, it will be further tested whether this prediction remains significant also after controlling for autocorrelations (i.e., the correlations between early and late process changes) and synchronous correlations (i.e., the correlations between early process and early outcome changes) [72]. The significance of the indirect effect of treatment on EDE-Q scores through the putative mediators will be determined using a bootstrap approximation with 5000 iterations to obtain biased-controlled confidence intervals. In case of multiple significant mediators, the independent contribution of these mediators will be further explored using multiple mediation models.

### Cost-effectiveness analysis

We will apply a cost-utility analysis (CUA). The results will be expressed as cost per Quality-adjusted Life Year (QALY). The economic evaluation will be undertaken from a societal perspective. Hence, all relevant effects and costs due to resource utilization within and outside the health care (direct costs) and costs due to production losses (indirect costs) will be included. To examine the cost-effectiveness of CBT-E compared to TAU the EQ-5D, the SF-36 and the TiC-P will be used.

The cost utility will be calculated as an incremental cost-effectiveness ratio (ICER) which is the ratio between the difference in costs and the difference in QALYs. The budget impact analysis (BIA) will be conducted from a health care payer perspective according to the ISPOR guidelines [57]. So, we will compare total health care costs when applying the intervention compared to the standard treatment for the target population in The Netherlands. Cost-effectiveness analyses will be performed using the ICEinfer package [73] within the R environment [74].

#### Dissemination

Results of the study will be presented at international scientific congresses and published in international scientific journals. Also, if applicable, the practical implications of the study outcome will be published in professional journals and can provide input for the Dutch Multidisciplinary Guideline for the Treatment of Eating Disorders. Moreover, depending on the outcome of the study, research findings will be used in the training of professionals.

### Ethical considerations

Ethical approval has been obtained from the Ethical Review Board of the Leiden University Medical Center. The Board of Directors at PsyQ agreed to support the execution of the study. The Boards of Directors of the three psychiatric regional centers that take part in the study also gave their consent. All participants will be extensively informed about the study, addressing confidentiality and the right to abort their participation at any time and without clarification; quitting the research program will by no means affect the subsequent course of treatment. No harm is expected from the intervention. In case of clinical deterioration (for any reason), the responsible clinical psychologist/psychiatrist can advise discontinuation of trial participation at any time. Written information will be given. When the patient is willing to continue, written consent is required. An independent physician is appointed, to whom subjects can address questions about the research before, during and after a study. The independent physician is not involved in the study itself.

## Discussion

In this study we assess the effectiveness of CBT-E. In addition to the assessment of changes in ED pathology and comorbid other psychopathology, we will also assess the differential cost-effectiveness of CBT-E compared to that of TAU. This is an important strength of this study because to our knowledge this has not yet been done. In a time were resources in health care are limited this question becomes more and more important. If CBT-E appears to be cost-effective for a broad range of ED patients, it would give the opportunity to offer treatment for a severe mental disorder with fewer resources, thereby increase the accessibility of specialized care for patients with an ED.

A large sample will be recruited and the sample is a clinically relevant one as it will be recruited among consecutive patients from three outpatient centers and few exclusion criteria are applied.

The follow-up will take place until 60 weeks after the end of treatment which gives the opportunity to look for long-term results. This is an important strength because there are few effectiveness studies in the field of EDs with long-term follow-up.

Low self-esteem, dysfunctional perfectionism and interpersonal problems have been identified in clinical practice and in research as possible factors for obstruction to change and progress. Therefore, these three factors are measured during this RCT before, during and after treatment to explore their possible predictive and/or mediating effects on treatment outcome.

There are, however, also some limitations to consider given the chosen research design.

Firstly, most of the measurements are conducted online which reduces research costs and maximizes the accessibility of participation. However, this could be a limitation because of the nonstandardized assessment situation and possible delay in collection of data between the moment the questionnaires are sent and the moment of completion.

Secondly, we include participants according to DSM-5 criteria while at the same time we use the SCID-I which is validated to assess the presence of an ED according to the DSM-IV. Because the SCID-I only covers AN, BN, and EDNOS-BED, skip rules were changed or omitted and parts of the *Eating Disorder Examination (EDE)* [31] were added in order to diagnose DSM-5 AN, BN, BED and OSFED. Although these adjustments have also been used in a recent epidemiological study [32], they have not yet been validated.

Thirdly, the present study uses TAU as the control condition; no alternative control conditions such as a no-treatment, waiting-list or placebo condition are included. This could also be a limitation especially when we do not find a difference in outcome between the two active treatment conditions. When no difference in outcome will be found, it will be difficult to determine to what extent the effect of treatment has to be ascribed to nonspecific factors, the effect of testing or the passage of time.

Fourthly, we designed our RCT as a superiority trial with enough statistical power to detect a difference in outcome between treatments (if present) with a medium effect size. However, it could be considered a limitation of the study that the power analysis was only based on detecting such difference in recovery rate because, with the therefore necessary 132 participants, only medium to large mediation effects can be tested with a power of 80% using bias-corrected bootstrapping procedures [75].

Fifthly, although we will determine treatment integrity, therapist competence in delivering the experimental intervention will not be assessed. However, because all participating CBT-E therapists will have been trained in CBT-E, and will have treated at least three ED patients with CBT-E under supervision before entering the trial, a sufficient level of competence may be assumed.

Finally, the sample size is too small to allow subgroup analyses and consequently the possible differential effectiveness of CBT-E for AN, BN, and EDNOS-BED cannot be assessed. Moreover, the changes in thresholds for AN and BN in DSM-5, the addition of BED as a new official diagnosis, and the redefinition of the remaining EDs from the DSM-IV EDNOS category into two categories complicate investigations of the differential effectiveness of CBT-E for diagnostic subcategories.

### Trial status

Recruiting.

## Additional files

Additional file 1: SPIRIT 2013 Checklist: recommended items to address in a clinical trial protocol and related documents. (DOC 125 kb)
Additional file 2: SPIRIT figure: schedule of enrollment, interventions, and assessments. (DOC 57 kb)

## Abbreviations

AN: Anorexia nervosa
BED: Binge eating disorder
BMI: Body Mass Index
BN: Bulimia nervosa
CBT: Cognitive behavioral therapy
CBT-E: Cognitive behavioral therapy-enhanced
DSM: *Diagnostic and Statistical Manual of Mental Disorders*
ED: Eating disorder
EDE-Q: The Eating Disorder Examination-Questionnaire
EDNOS: Eating disorder not otherwise specified
EQ-5D: EuroQoL five dimensions questionnaire
F-MPS: Frost Multidimensional Perfectionism Scale
IAT: Implicit Association Test Self-esteem
IIP-32: Inventory of Interpersonal Problems
MASQ: Mood and Anxiety Symptom Questionnaire
OSFED: Other specified feeding or eating disorder
RSE: Rosenberg Self-Esteem Scale
SCID-I: Structured Clinical Interview for DSM Axis-I Disorders
SF-36: Short Form Health Survey
SF-HLQ: Short Form – Health and Labour Questionnaire
Tic-P: Trimbos/iMTA Questionnaire for Costs associated with Psychiatric Illness
USFED: Unspecified feeding or eating disorder
WSQ: Web Screening Questionnaire for common mental disorders
