# Supplementary material for: Effectiveness of enhanced cognitive behavioral therapy (CBT-E) for eating disorders: study protocol for a randomized controlled trial
Source: Trials. 2016 Dec 3;17:573. doi: 10.1186/s13063-016-1716-3 (PMC5135785; doi:10.1186/s13063-016-1716-3)
Supplement: Additional file 2: — SPIRIT figure: schedule of enrollment, interventions, and assessments. (DOC 57 kb) [file 13063_2016_1716_MOESM2_ESM.doc]

**SPIRIT Figure:** Schedule of enrolment, interventions, and assessments

|  |  | **STUDY PERIOD** | | | | |
| --- | --- | --- | --- | --- | --- | --- |
|  | **Enrolment** | **Allocation** | **Post-allocation** | | **Follow-up** | |
| **TIMEPOINT*** | ***-t_1_*** | **0** | ***t_1_*** | ***t_2_*** | ***t_3_*** | ***t_4_*** |
| **ENROLMENT:** |  |  |  |  |  |  |
| **Eligibility screen** | X |  |  |  |  |  |
| **Informed consent** | X |  |  |  |  |  |
| **Allocation** |  | X |  |  |  |  |
| **INTERVENTIONS:** |  |  |  |  |  |  |
| ***CBT-E*** |  |  |  |  | X |  |
| ***TAU*** |  |  |  | ** |  |  |
| **ASSESSMENTS:***** |  |  |  |  |  |  |
| ***SCID-I*** | X |  |  | X |  | X |
| ***EDE-Q*** |  | X | X | X | X | X |
| ***WSQ*** |  | X | X | X | X | X |
| ***MASQ*** |  | X | X | X | X | X |
| ***EQ-5D*** |  | X | X | X | X | X |
| ***SF-36*** |  | X | X | X | X | X |
| ***TiC-P*** |  | X | X | X | X | X |
| ***RSE*** |  | X | X | X | X | X |
| ***IAT*** |  | X |  | X |  |  |
| ***F-MPS*** |  | X | X | X | X | X |
| ***IIP-32*** |  | X | X | X | X | X |

**<Figure Legend>**

*** 0 = Baseline, *t_1_* = Week 6, *t_2_* = End of treatment Week 20, *t_3_* = Follow up Week 40, *t_4_* = Follow Up Week 80.**

**** TAU, length and intensity of the therapy can vary**

***** SCID-I = Structured Clinical Interview for DSM Axis-I disorders; EDE-Q = The Eating Disorder Examination- Questionnaire; WSQ = Web screening Questionnaire for common mental disorders; MASQ = Mood and Anxiety Questionnaire; EQ-5D = EuroQoL; SF-36 = Short Form Health Survey; TiC-P = Trimbos/iMTA Questionnaire for Costs associated with Psychiatric Illness; RSE = Rosenberg Self-Esteem scale;
IAT = Implicit Association Test Self-Esteem; F-MPS = Frost Multidimensional Perfectionism Scale;
IIP‑32 = Inventory of Interpersonal Problems**
